# Region-specific brain metabolite reference estimates and exploratory age-related changes in cognitively intact older adults: a preliminary deep-learning ^1^H-MRS study

**DOI:** 10.3389/fnagi.2026.1896159

**Published:** 2026-08-06

**Authors:** Young-Ah Choi, Hyeong Hun Lee, Dong-Kyu Jang, Junghyeon Park, Si Won Yang, Min-Wook Kim

**Affiliations:** 1 Department of Rehabilitation Medicine, Incheon St. Mary’s Hospital, College of Medicine, The Catholic University of Korea, Seoul, Republic of Korea; 2 Research and Development Center, METLiT Inc., Seoul, Republic of Korea; 3 Department of Neurosurgery, Incheon St. Mary’s Hospital, College of Medicine, The Catholic University of Korea, Seoul, Republic of Korea

**Keywords:** aging, deep learning, default mode network, magnetic resonance spectroscopy, neurochemistry, posterior cingulate cortex, reference values

## Abstract

**Introduction:**

Normative brain metabolite references are essential for evaluating metabolic change in rehabilitation patients with stroke, traumatic brain injury, or cognitive decline. We provide preliminary, cohort-specific reference estimates in cognitively healthy older adults using proton magnetic resonance spectroscopy (^1^H-MRS) and examine regional and age-related differences.

**Methods:**

Twenty healthy older adults (10 males, 10 females; mean age 71.0 ± 9.5 years, range 56–84) underwent single-voxel *^1^H*-MRS at 3T in three regions: dorsal anterior cingulate cortex (DACC), posterior cingulate cortex (PCC), and occipital cortex (OC). A Bayesian deep-learning convolutional neural network quantified 14 metabolite ratios normalized to total creatine (*tCr*). Because regions were acquired within each participant (20 independent observations), regional differences were evaluated primarily with the within-subject Friedman test (Kendall’s *W*), with Kruskal–Wallis as a secondary analysis; Benjamini–Hochberg false discovery rate (FDR) correction was applied across the 14 metabolites. Age– and MMSE–metabolite associations used Spearman’s rank correlation with FDR applied both across all 42 tests and within each region.

**Results:**

Nine of 14 metabolites showed significant regional differences (seven also surviving the primary Friedman test after FDR correction), with glycerophosphocholine (GPC)/tCr showing the largest effect (Friedman *W* = 0.82; Kruskal–Wallis ε^2^ = 0.55). γ-aminobutyric acid (GABA)/tCr was highest in the OC, whereas myo-inositol (mI) and choline compounds were highest in the DACC. In exploratory age analyses, the PCC showed negative correlations for aspartate (Asp)/tCr (ρ = –0.61, *p* = 0.004) and glutathione (GSH)/tCr (ρ = –0.59, *p* = 0.006), both surviving region-wise FDR correction (*q* = 0.044), with glutamate (Glu)/tCr nominal (ρ = –0.45) and total N-acetylaspartate (tNAA)/tCr at trend level (ρ = –0.39); the DACC showed positive correlations for glucose (Glc)/tCr (ρ = +0.60) and lactate (Lac)/tCr (ρ = +0.50). No association survived the more conservative correction across all 42 tests. The OC showed no significant age associations.

**Conclusion:**

This study established preliminary, cohort-specific multi-regional *^1^H*-MRS reference estimates for cognitively intact older adults, with robust regional profiles and large effect sizes. The PCC showed exploratory age-related change—most consistently a coordinated aspartate–glutathione decline—requiring confirmation in larger cohorts, whereas the OC emerged as a candidate metabolically stable region.

## 1 Introduction

Proton magnetic resonance spectroscopy (^1^H-MRS) is a non-invasive neuroimaging technique that enables *in vivo* quantification of brain metabolites, providing biochemical information complementary to conventional structural MRI (Oz et al., 2014). Major metabolites measurable by ^1^H-MRS include N-acetylaspartate (NAA), a marker of neuronal integrity; myo-inositol (mI), an astrocytic marker; choline compounds (tCho), reflecting membrane turnover; glutamate/glutamine (Glx), excitatory neurotransmitters; and γ-aminobutyric acid (GABA), the primary inhibitory neurotransmitter (Barker et al., 1993). These metabolites serve as sensitive biomarkers for various neurological conditions encountered in rehabilitation medicine, including stroke, traumatic brain injury (TBI), and neurodegenerative diseases.

Conventional metabolite quantification from ^1^H-MRS spectra has historically relied on linear-combination model fitting using prior-knowledge basis sets, as implemented in widely used packages such as LCModel (Provencher, 2001). Although these approaches have become the field standard, their performance is known to degrade under low signal-to-noise ratio (SNR), baseline distortion, and line broadening, and the Cramér–Rao lower bound (CRLB) returned by such fits tends to underestimate the true quantification uncertainty in non-ideal acquisitions (Kreis, 2016). Deep-learning (DL)-based quantification has recently emerged as an alternative that learns a direct nonlinear mapping from the input spectrum to metabolite concentrations using large, physics-informed simulated training corpora, thereby bypassing iterative model fitting (Gurbani et al., 2019; Lee and Kim, 2019). These frameworks have demonstrated robust quantification across a broad range of SNR and linewidth conditions and reduced dependence on user-specified fitting parameters, although further validation against established methods remains warranted (Lee and Kim, 2020). In particular, Bayesian convolutional neural networks (CNNs) with Monte Carlo (MC) dropout further provide well-calibrated, per-spectrum uncertainty estimates, offering a principled substitute for CRLB-based quality control (Lee and Kim, 2022).

Accurate interpretation of metabolic abnormalities in patient populations requires reliable normative reference values established from healthy controls. While several studies have reported ^1^H-MRS normative data (Pouwels and Frahm, 1998; Haga et al., 2009; Eylers et al., 2016), most have examined limited brain regions or narrow age ranges, and population-specific normative references remain scarce. Given documented ethnic and demographic differences in brain morphometry and potentially in neurochemistry (Raz et al., 2010), establishing region-specific normative databases tailored to the target population is of particular importance for accurate clinical interpretation.

The posterior cingulate cortex (PCC), a core hub of the default mode network (DMN), has received considerable attention as one of the earliest regions affected in Alzheimer’s disease and age-related cognitive decline (Buckner et al., 2008). Understanding normal age-related metabolic trajectories in the PCC, as well as in other functionally distinct regions such as the dorsal anterior cingulate cortex (DACC, involved in executive function and salience processing) and the occipital cortex (OC, primary visual processing), is essential for distinguishing pathological from physiological changes.

Against this backdrop, four specific gaps motivate the present work. First, normative references acquired specifically in healthy older adults—the age stratum most relevant to neurodegenerative disease screening—remain limited, and existing datasets often conflate adults spanning several decades. Second, few studies have reported simultaneous, within-subject measurements across functionally distinct regions; our acquisition of the dorsal anterior cingulate cortex (DACC), posterior cingulate cortex (PCC), and occipital cortex (OC) in the same cohort permits direct regional comparison while controlling for inter-individual variability. Third, to our knowledge no prior normative study has applied a DL-based quantification pipeline, despite its demonstrated robustness at the low SNR and broadened linewidth frequently encountered in elderly spectra (Lee and Kim, 2019; Lee and Kim, 2020; Lee and Kim, 2022). Fourth, population-specific normative values for Korean older adults are presently unavailable, even though ethnic and demographic differences in brain morphometry and potentially in neurochemistry have been documented (Raz et al., 2010). The present study was therefore designed to establish region-specific ^1^H-MRS normative reference ranges for the DACC, PCC, and OC in healthy older adults using a validated Bayesian CNN quantification framework, and to characterize age-related metabolic variation within this cohort.

The present study aimed to: (1) establish a preliminary multi-regional normative metabolite dataset in cognitively intact older adults using deep learning–based ^1^H-MRS; (2) characterize regional metabolite distribution profiles; and (3) explore age-related metabolite changes across three functionally distinct cortical regions.

## 2 Materials and methods

### 2.1 Participants

Twenty-one community-dwelling healthy older adults were prospectively recruited. One participant (S11) was excluded due to dropout during the study, yielding a final sample of 20 participants (10 males, 10 females; mean age 71.0 ± 9.5 years, range 56–84 years). Inclusion criteria were: (1) age ≥ 55 years; (2) Mini Mental State Examination (MMSE) score ≥ 24, indicating normal cognitive function; (3) no history of neurological or psychiatric disorders; (4) no history of brain surgery or significant head trauma; and (5) no contraindications to MRI. All participants were right-handed.

The mean MMSE score was 28.2 ± 1.6 (range 24–30, median 28.5), confirming preserved cognitive function across the cohort. Body mass index (BMI) was 25.5 ± 2.6 kg/m^2^ (range 20.5–32.0). There were no significant differences between males and females in age (*p* = 0.85), BMI (*p* = 0.91), or MMSE scores (*p* > 0.999) (Table 1). The study was approved by the Institutional Review Board of Incheon St. Mary’s Hospital (IRB No. OC25EISI0002), and written informed consent was obtained from all participants.

**TABLE 1:** Demographic and clinical characteristics of participants (*n* = 20).

Variable	Total (*n* = 20)	Male (*n* = 10)	Female (*n* = 10)	*U*	*p*
Age (years)	71.0 ± 9.5	71.7 ± 9.6	70.2 ± 9.8	53	0.849
Height (cm)	159.1 ± 8.3	165.1 ± 4.8	153.1 ± 6.6	96	0.001
Weight (kg)	64.9 ± 10.0	69.5 ± 7.3	60.3 ± 10.6	80	0.026
BMI (kg/m^2^)	25.5 ± 2.6	25.5 ± 1.9	25.6 ± 3.3	52	0.910
MMSE	28.2 ± 1.6	28.4 ± 1.2	28.1 ± 2.0	50.5	1.000

Values are presented as mean ± SD. Mann–Whitney *U* test for sex comparisons. BMI, body mass index; MMSE, Mini Mental State Examination. *p < 0.05, **p < 0.01.

The sample size of *n* = 20 was selected on the basis of precedent set by foundational normative *^1^H*-MRS studies establishing region-specific reference values: Pouwels and Frahm (1998) reported a canonical atlas using *n* = 33 across nine voxels, and Minati et al. (2010) derived concentrations across six regions with *n* = 15. For region-specific reference estimation, samples of 15–30 controls per region are routinely considered sufficient to characterize central tendency and inter-individual variability. Because the three regions were measured within each participant, the design comprised 20 independent observations (60 within-subject spectra), not 60 independent samples. The cohort spanned three age bands—56–65 years (*n* = 7), 66–75 years (*n* = 4), and 76–84 years (*n* = 9)—and is explicitly framed as a preliminary, pilot dataset. A *post-hoc* analysis indicated that approximately 29 participants would be required to detect a correlation of ρ = 0.50 at 80% power; age-effect estimates are therefore reported as hypothesis-generating and deferred to adequately powered confirmatory cohorts.

### 2.2 MRS data acquisition

All MRS data were acquired on a 3T MRI scanner (MAGNETOM Vida; Siemens Healthineers, Erlangen, Germany) equipped with a 64-channel head/neck coil. Single-voxel ^1^H-MRS was performed using a point-resolved spectroscopy (PRESS) sequence with short echo time (TR/TE = 2000/35 ms). Voxels were placed in three regions of interest: (1) dorsal anterior cingulate cortex (DACC), (2) posterior cingulate cortex (PCC), and (3) occipital cortex (OC). Voxel size was approximately 8 cm^3^ for each region. A total of 32 signal averages were acquired for water-suppressed spectra and 8 averages for water-unsuppressed reference spectra (used for eddy-current correction and absolute referencing), with a spectral bandwidth of 2000 Hz and 2048 data points. Water suppression was achieved with the vendor-supplied WET (water suppression enhanced through *T1* effects) scheme, and B_0_ homogeneity within each voxel was optimized using the scanner’s automated 3-D shimming routine prior to each acquisition. The acquisition time per voxel was less than 2 min, resulting in a total MRS acquisition time of less than 6 min for all three regions.

**FIGURE 1:**
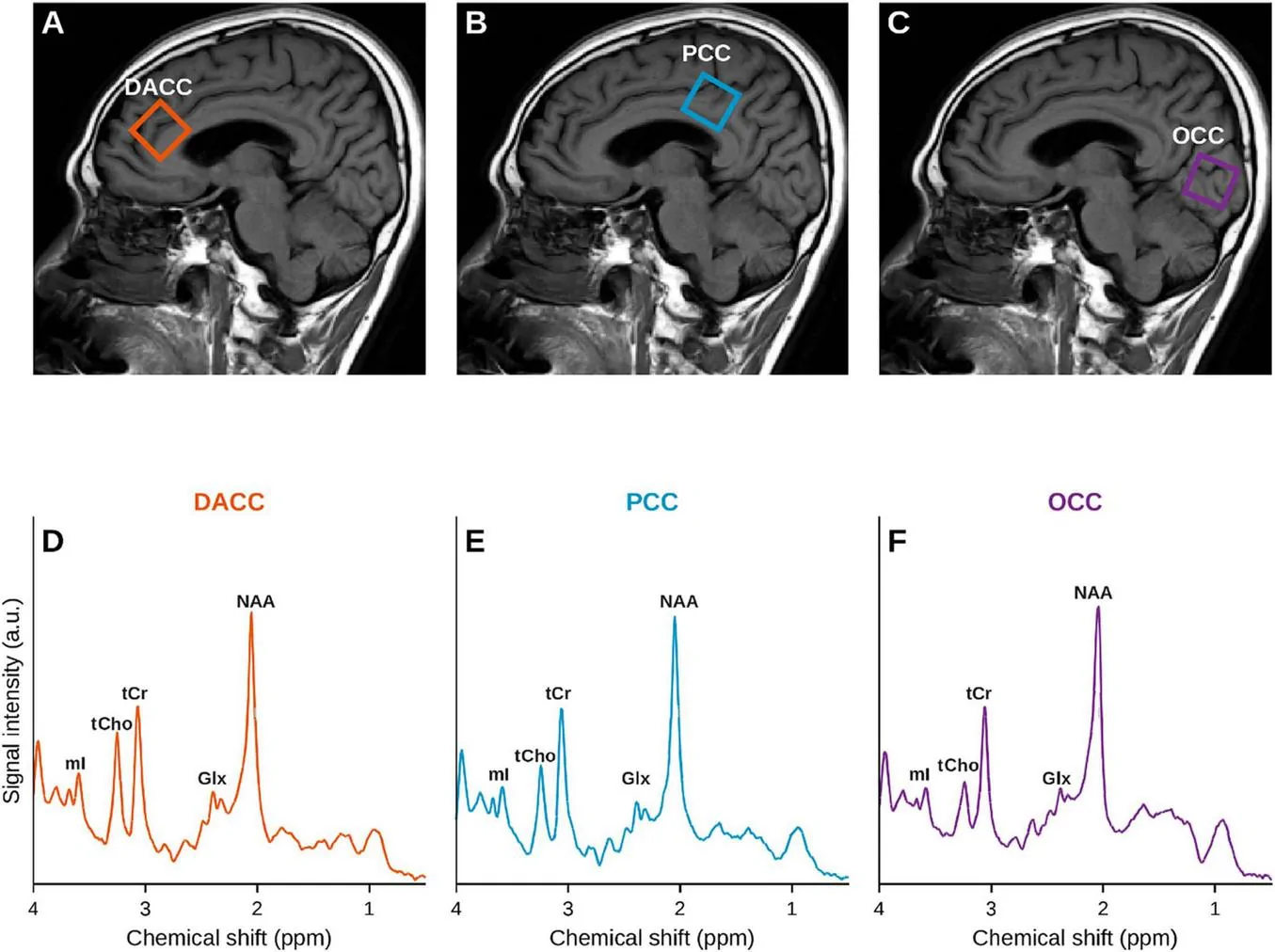
Representative MRI images with voxel-of-interest (VOI) placement and corresponding ^1^H-MRS spectra. **(A–C)** T_2_-weighted sagittal MRI showing VOI locations for **(A)** dorsal anterior cingulate cortex (DACC), **(B)** posterior cingulate cortex (PCC), and **(C)** occipital cortex (OC). **(D–F)** Representative ^1^H-MRS spectra acquired from each region with major metabolite peak assignments. NAA, N-acetylaspartate; tCr, total creatine; tCho, total choline; mI, myo-inositol; Glx, glutamate + glutamine; GABA, γ-aminobutyric acid.

Voxel placement was performed by a single MRS-experienced operator following standardized anatomical landmarks defined *a priori* (DACC: pregenual midline; PCC: midline precuneus immediately posterior to the splenium; OC: medial occipital gray matter centered on the calcarine sulcus) to ensure consistency across subjects. Sagittal, axial, and coronal *T2*-weighted images were used for voxel localization. Representative voxel placements and corresponding MRS spectra are shown in Figure 1.

### 2.3 Spectral quantification

Metabolite quantification was performed using a convolutional neural network (CNN)-based deep learning approach developed by Lee and Kim (2019, 2020, 2022). The CNN was designed to map *in vivo* brain spectra—typically degraded by low signal-to-noise ratio (SNR), line broadening, and spectral baseline—into noise-free, line-narrowed, baseline-removed intact metabolite spectra. The network was trained on 10 million *in silico* brain spectra with wide ranges of SNR (3 – 120) and linewidth (3–30 Hz), and has been validated against LCModel (Provencher, 2001) on *in vivo* human brain data with demonstrated robustness to low-SNR conditions (Lee and Kim, 2019). The Bayesian CNN extension with Monte Carlo dropout sampling enables simultaneous metabolite quantification and measurement uncertainty estimation (Lee and Kim, 2022). The CNN quantified concentrations for 21 metabolites. For this analysis, 14 metabolites with reliable quantification were selected: N-acetylaspartate (NAA), total NAA (tNAA = NAA + NAAG), aspartate (Asp), glutamate (Glu), glutamine (Gln), Glx (Glu + Gln), glucose (Glc), myo-inositol (mI), total choline (tCho = GPC + PCh), glycerophosphocholine (GPC), phosphocholine (PCh), γ-aminobutyric acid (GABA), glutathione (GSH), and lactate (Lac).

All metabolite concentrations were normalized to total creatine (tCr = Cr + PCr) as an internal reference. The deep-learning pipeline produced two complementary quality metrics: (i) a spectrum-level goodness-of-fit between the input and the network-reconstructed model spectrum, quantified by the coefficient of determination (R^2^); and (ii) a per-metabolite Bayesian relative uncertainty from Monte-Carlo-dropout sampling of the Bayesian CNN, expressed as a percentage of the estimated amplitude and analogous in role to the Cramér–Rao lower bound (CRLB) of conventional fitting (Lee and Kim, 2022). Spectra with *R*^2^ < 0.70 were flagged for review; 3 of 60 spectra (5.0%), all in the DACC (S06: *R*^2^ = 0.58; S14: *R*^2^ = 0.69; S17: *R*^2^ = 0.54), fell below this threshold. To preserve the full within-subject design, all 60 spectra were included in the primary analyses, and these three flagged DACC spectra were removed only in a pre-specified sensitivity analysis (Table 2), which reproduced the principal regional findings (tCho being the only comparison to attenuate to non-significance). Per-metabolite Bayesian relative uncertainties are reported in Supplementary Table 6 and Supplementary Figure 1: well-resolved metabolites (NAA, tNAA, Glu, Glx, mI, tCho, Glc) had low median uncertainty (≈5% or less), whereas low-concentration metabolites (GABA, Asp, GSH, Lac) had higher uncertainty and are interpreted with corresponding caution. No separate LCModel-derived CRLB was computed, because quantification did not use linear-combination fitting; the R^2^ ≥ 0.70 flag follows conventions in the deep-learning MRS literature (Lee and Kim, 2019), and preprocessing and quality assessment were consistent with consensus single-voxel MRS recommendations (Wilson et al., 2019; Near et al., 2021).

**TABLE 2:** Sensitivity analysis: regional comparisons with and without low *R*^2^ DACC spectra.

Metabolite	*H* (full)	*p* (full)	Sig.	*H* (filtered)	*p* (filtered)	Sig.	Conclusion
*NAA*	9.42	0.009	**	9.68	0.008	**	Consistent
*tNAA*	13.04	0.001	**	13.19	0.001	**	Consistent
*Asp*	16.84	< 0.001	***	15.89	< 0.001	***	Consistent
*Glc*	9.61	0.008	**	8.65	0.013	*	Consistent
*mI*	9.34	0.009	**	6.17	0.046	*	Consistent
*tCho*	7.45	0.024	*	5.23	0.073	ns	Attenuated (n.s.)
*GPC*	33.56	< 0.001	***	30.32	< 0.001	***	Consistent
*GABA*	15.87	< 0.001	***	17.20	< 0.001	***	Consistent
*Lac*	11.30	0.004	**	8.76	0.013	*	Consistent

Sensitivity analysis of the regional comparison (independent-samples Kruskal–Wallis *H*, consistent with the secondary analysis in Supplementary Table 2), with and without the three DACC spectra with *R*^2^ < 0.70. Full analysis: all 20 participants per region. Filtered: DACC *n* = 17 (excluding 3 spectra with *R*^2^ < 0.70); PCC and OC *n* = 20.

*p < 0.05,

**p < 0.01,

***p < 0.001.

The Bayesian CNN quantification framework employed here was developed, trained, and validated in prior work (Lee and Kim, 2019, 2022); the present study is not a model-development study but an application of this validated pipeline to a new cohort. The earlier validation established (i) agreement with LCModel on *in vivo* human brain spectra, with the network showing lower cross-SNR variability (≈10% or less) than LCModel (Lee and Kim, 2019), and (ii) well-calibrated per-spectrum Bayesian uncertainty via Monte-Carlo dropout that tracked ground-truth error at least as closely as LCModel CRLB in degraded *in vivo* spectra (Lee and Kim, 2022). A concise summary of this published cross-method validation is provided in Supplementary methods 7.

### 2.4 Statistical analysis

All statistical analyses were performed in Python 3.12 with SciPy (v1.12). Given the modest sample size and non-normal distribution of several ratios, non-parametric methods were used throughout. Because the three regions (DACC, PCC, OC) were measured within each participant, regional metabolite comparisons were performed primarily with the Friedman repeated-measures test (Friedman, 1937), which accounts for within-subject dependence, using Kendall’s W as the standardized effect size and Dunn’s test with Benjamini–Hochberg correction for *post-hoc* pairwise comparisons. The independent-samples Kruskal–Wallis H test (with ε^2^ effect size and Bonferroni-corrected Mann–Whitney U *post-hoc* tests) was retained as a secondary, concordance analysis (Supplementary Table 2). Benjamini–Hochberg FDR correction (Benjamini and Hochberg, 1995) was applied across the 14 metabolites for both omnibus families. Kruskal–Wallis effect sizes were computed as ε^2^ = (H – k + 1)/(N – k) (Tomczak and Tomczak, 2014).

Age–metabolite correlations were assessed using Spearman’s rank correlation coefficient (ρ) for each metabolite within each region (42 tests: 14 metabolites × 3 regions). Benjamini–Hochberg FDR correction was applied both across the full family of 42 tests and, separately, within each region (three families of 14 tests), the latter reflecting the anatomically distinct nature of the three acquisitions; both schemes are reported (Supplementary Table 1). Bootstrap 95% confidence intervals (10,000 iterations) were computed for correlations with uncorrected *p* < 0.05. Sex differences were examined with Mann–Whitney *U* tests, and MMSE–metabolite associations analogously with FDR correction (Supplementary Table 5). Sensitivity analyses excluded the three DACC spectra with *R*^2^ < 0.70 (*n* = 17 for DACC; *n* = 20 for PCC and OC). Reference estimates were defined as mean ± 2 SD (CLSI EP28-A3c, 2010; Solberg, 2004), with normality assessed by the Shapiro–Wilk test and distribution-free 2.5th–97.5th-percentile intervals additionally reported for non-normal ratios (Supplementary Tables 3, 4). The significance level was α = 0.05.

## 3 Results

### 3.1 Participant characteristics

Demographic and clinical characteristics of the 20 participants are summarized in Table 1. The cohort included 10 males and 10 females across a wide age range (56–84 years; 56–65 *n* = 7, 66–75 *n* = 4, 76–84 *n* = 9). All participants had preserved cognition (MMSE ≥ 24; mean 28.2 ± 1.6). Age and MMSE were not significantly correlated (ρ = –0.28, *p* = 0.233). A total of 60 within-subject spectra (20 participants × 3 regions; 20 independent observations) were acquired, of which 57 (95.0%) met the *R*^2^ ≥ 0.70 quality threshold.

### 3.2 Regional metabolite profiles

Table 3 presents metabolite-to-*tCr* ratios across the three regions, analyzed primarily with the within-subject Friedman test. Nine of 14 metabolites showed significant regional differences that survived Benjamini–Hochberg FDR correction under the secondary Kruskal–Wallis analysis, and seven remained significant under the more conservative within-subject Friedman analysis (tNAA, Asp, Glc, mI, tCho, GPC, GABA; NAA at *q* = 0.061 and Lac at *q* = 0.121). GPC/*tCr* showed the largest effect (Friedman *W* = 0.82; *ε^2^* = 0.55), with a clear anterior-to-posterior gradient (DACC 0.21 ± 0.07 > PCC 0.14 ± 0.04 > OC 0.09 ± 0.03). GABA/*tCr* was highest in the OC (0.25 ± 0.07) versus DACC (0.18 ± 0.07) and PCC (0.17 ± 0.05). NAA/*tCr* and tNAA/*tCr* showed a posterior-dominant gradient with highest concentrations in the OC, consistent with established literature.

**TABLE 3:** Metabolite-to-tCr ratios by brain region with regional comparison statistics.

Met/*tCr*	DACC (*n* = 20)	PCC (*n* = 20)	OC (*n* = 20)	χ^2^(2)	*p*	*q* (FDR)	*W*
*NAA*	1.33 ± 0.31	1.43 ± 0.19	1.64 ± 0.32	6.70	0.035	0.061	0.17
*tNAA*	1.44 ± 0.23	1.54 ± 0.17	1.74 ± 0.28	10.00	0.007	0.016	0.25
*Asp*	0.26 ± 0.06	0.21 ± 0.03	0.29 ± 0.07	12.70	0.002	0.008	0.32
*Gln*	0.35 ± 0.15	0.29 ± 0.06	0.35 ± 0.07	2.50	0.287	0.334	0.06
*Glu*	1.17 ± 0.25	1.19 ± 0.15	1.18 ± 0.19	1.90	0.387	0.387	0.05
*Glx*	1.52 ± 0.31	1.49 ± 0.18	1.53 ± 0.24	1.90	0.387	0.387	0.05
*Glc*	0.36 ± 0.13	0.25 ± 0.08	0.33 ± 0.14	11.20	0.004	0.010	0.28
*mI*	1.17 ± 0.20	1.03 ± 0.16	1.01 ± 0.18	11.70	0.003	0.010	0.29
*tCho*	0.52 ± 0.25	0.39 ± 0.12	0.34 ± 0.18	8.40	0.015	0.030	0.21
*GPC*	0.21 ± 0.07	0.14 ± 0.04	0.09 ± 0.03	32.70	< 0.001	<0.001	0.82
*PCh*	0.31 ± 0.20	0.25 ± 0.10	0.25 ± 0.16	3.70	0.157	0.200	0.09
*GABA*	0.18 ± 0.07	0.17 ± 0.05	0.25 ± 0.07	13.30	0.001	0.008	0.33
*GSH*	0.14 ± 0.07	0.15 ± 0.05	0.11 ± 0.03	4.90	0.086	0.121	0.12
*Lac*	0.27 ± 0.19	0.11 ± 0.06	0.14 ± 0.09	4.90	0.086	0.121	0.12

Values are mean ± SD. Primary analysis: within-subject Friedman repeated-measures test. χ^2^(2), Friedman statistic (2 df); p, uncorrected; q, Benjamini–Hochberg FDR-adjusted p across 14 metabolites; W, Kendall’s coefficient of concordance (effect size: small ≈0.1, medium ≈0.3, large ≈0.5). DACC, dorsal anterior cingulate cortex; PCC, posterior cingulate cortex; OC, occipital cortex.

**FIGURE 2:**
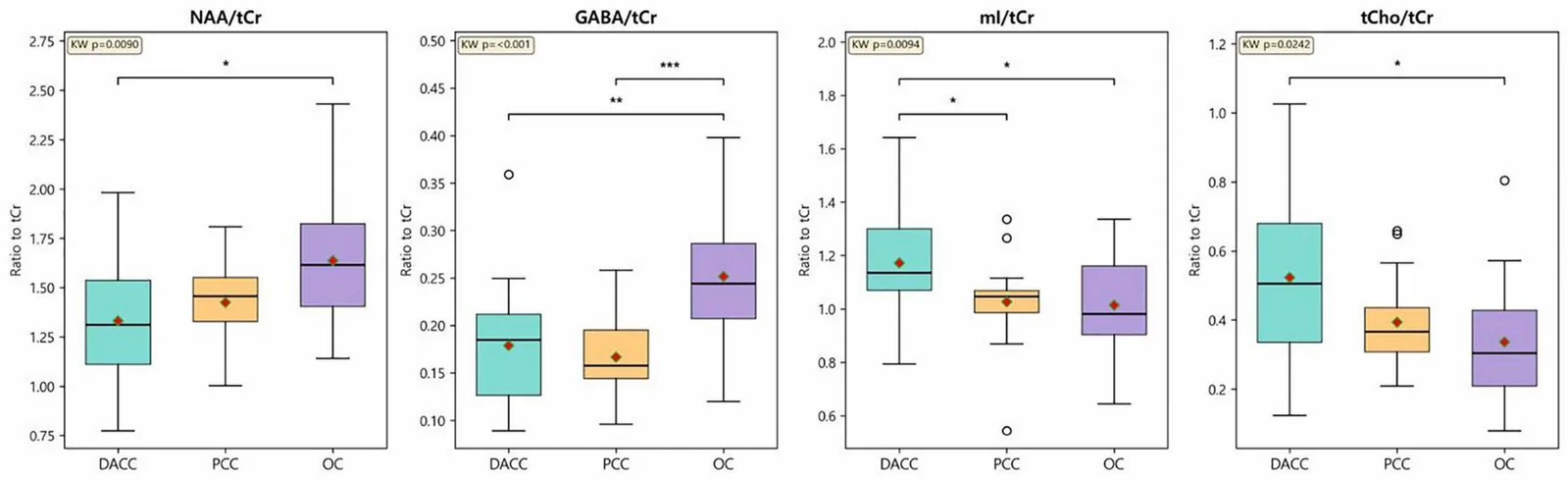
Regional distribution of key metabolite-to-tCr ratios across DACC, PCC, and OC. Box plots show median (line), mean (diamond), interquartile range (box), and 1.5 × interquartile range (whiskers); points beyond 1.5 × IQR are plotted individually as outliers. *p < 0.05, **p < 0.01, ***p < 0.001.

Myo-inositol (mI/tCr) was elevated in the DACC (1.17 ± 0.20) relative to the PCC (1.03 ± 0.16) and OC (1.01 ± 0.18). Total choline (tCho/tCr) followed a similar anterior-dominant pattern (DACC > OC). Aspartate showed an inverted pattern, with lowest concentrations in the PCC (0.21 ± 0.03) relative to the DACC (0.26 ± 0.06) and OC (0.29 ± 0.07). Lactate was higher in the DACC than the PCC. *Post-hoc* pairwise comparisons are indicated in Figure 2.

### 3.3 Age-related metabolite changes

**FIGURE 3:**
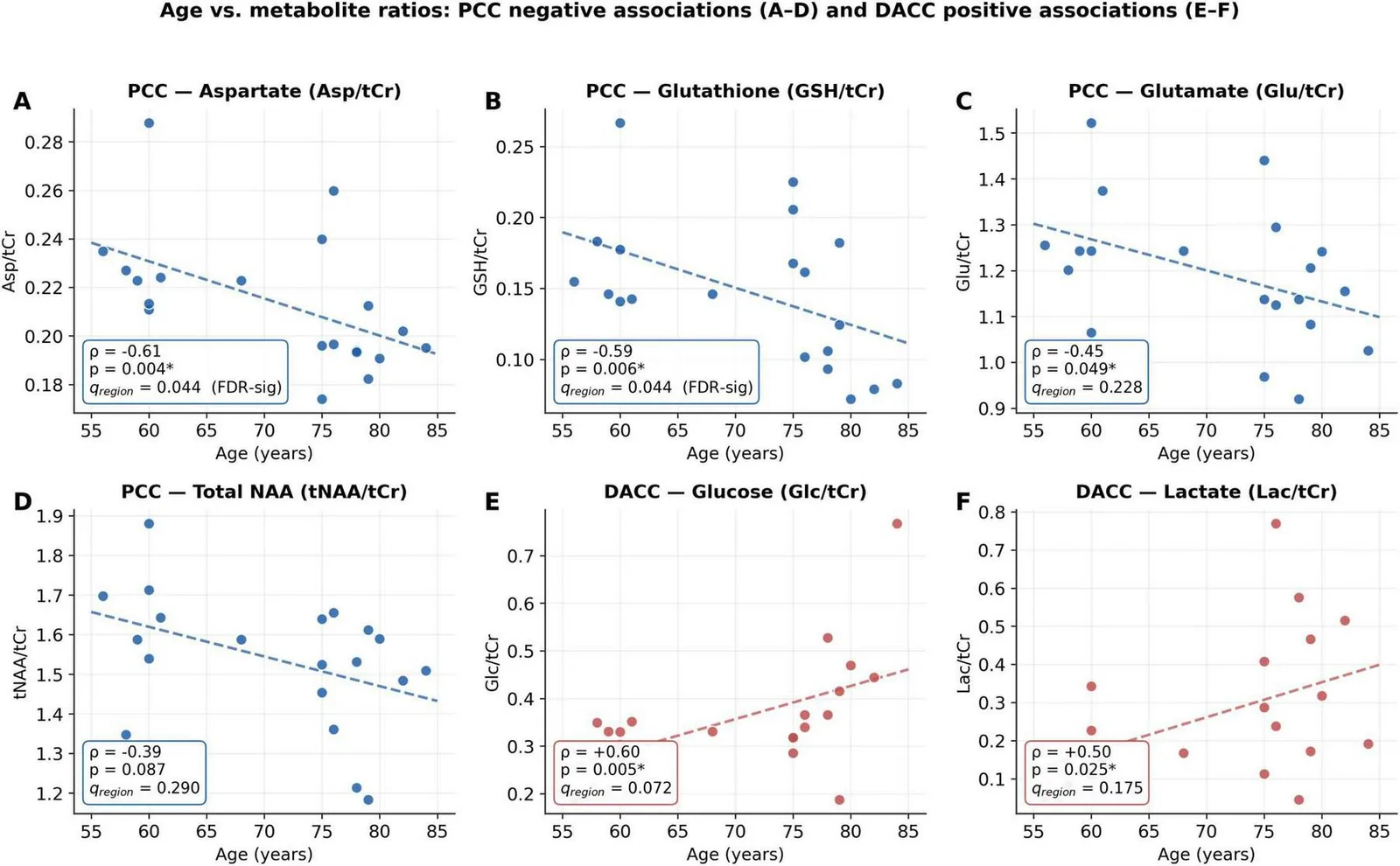
Age versus metabolite-to-*tCr* ratios for metabolites with the strongest age associations, shown as a 2 × 3 panel grid for legibility. **(A–C)** PCC negative associations (aspartate, glutathione, glutamate). **(D–F)** PCC total NAA (trend) and DACC positive associations (glucose, lactate). Dashed lines are linear-regression trends for visualization only; inference is based on Spearman rank correlation. Spearman ρ, uncorrected *p*, and region-wise FDR-adjusted *q* are annotated. PCC aspartate and glutathione survived region-wise FDR correction (*q* = 0.044); no association survived correction across all 42 tests.

Table 4 presents Spearman correlations between age and metabolite ratios. In exploratory analyses, the PCC showed negative age correlations for Asp/*tCr* (ρ = –0.61, *p* = 0.004), GSH/*tCr* (ρ = –0.59, *p* = 0.006), and Glu/*tCr* (ρ = –0.45, *p* = 0.049), with a trend for tNAA/*tCr* (ρ = –0.39, *p* = 0.087) and mI/*tCr* (ρ = –0.37, *p* = 0.104). The DACC showed positive correlations for Glc/*tCr* (ρ = +0.60, *p* = 0.005) and Lac/*tCr* (ρ = +0.50, *p* = 0.025) (Figure 3).

**TABLE 4:** Spearman correlation coefficients between age and metabolite-to-tCr ratios by brain region.

VOI	*DACC*		*PCC*		*OC*	
Met/tCr	ρ	*p*	ρ	*p*	ρ	*p*
*NAA*	+0.02	0.929	–0.32	0.168	+0.16	0.490
*tNAA*	+0.10	0.687	–0.39	0.087	+0.11	0.632
*Asp*	+0.17	0.486	–0.61	0.004**	+0.32	0.162
*Gln*	+0.39	0.088	+0.15	0.515	–0.02	0.942
*Glu*	+0.25	0.280	–0.45	0.049*	+0.12	0.601
*Glx*	+0.30	0.205	–0.24	0.308	+0.13	0.587
*Glc*	+0.60	0.005**	+0.22	0.350	+0.33	0.152
*mI*	–0.01	0.972	–0.37	0.104	+0.23	0.338
*tCho*	+0.26	0.277	–0.26	0.274	+0.16	0.510
*GPC*	+0.10	0.683	+0.12	0.603	+0.15	0.521
*PCh*	+0.29	0.220	–0.34	0.144	+0.12	0.601
*GABA*	+0.31	0.184	–0.29	0.219	+0.06	0.786
*GSH*	+0.21	0.385	–0.59	0.006**	+0.04	0.879
*Lac*	+0.50	0.025*	–0.11	0.632	+0.33	0.155

Spearman’s rank correlation coefficient (ρ); *p*, uncorrected.

*p < 0.05,

**p < 0.01. Under region-wise Benjamini–Hochberg FDR correction (14 tests per region), PCC Asp/*tCr* and GSH/*tCr* survived (*q* = 0.044); no association survived correction across all 42 tests (all *q* > 0.08). See Supplementary Table 1 for both FDR schemes.

When Benjamini–Hochberg FDR correction was applied within each region (three families of 14 tests, reflecting the anatomical independence of the acquisitions), the PCC Asp/*tCr* and GSH/*tCr* associations survived correction (both *q* = 0.044), whereas PCC Glu/*tCr* (*q* = 0.228), PCC tNAA/*tCr* (*q* = 0.290), and DACC Glc/*tCr* (*q* = 0.072) did not. Under the more conservative correction across all 42 tests, no association reached significance (smallest *q* = 0.088). These age associations are therefore reported as exploratory and hypothesis-generating, with the coordinated PCC aspartate–glutathione decline the most robust signal. Notably, the OC demonstrated no statistically significant age associations across the 56–84-year range: none of the 14 metabolites showed a significant age correlation (all | ρ| < 0.35, all *p* > 0.15).

### 3.4 Sex differences

No statistically significant sex differences were observed for any metabolite ratio in any brain region (all Mann–Whitney *U* test *p* > 0.05). In the PCC, Asp/tCr, Gln/tCr, and Glx/tCr showed trend-level sex differences (*p* = 0.07–0.08 for each) with slightly higher values in males, but these did not reach statistical significance. The absence of sex effects supports the pooling of male and female data for normative reference establishment in this age group.

### 3.5 Sensitivity analysis

In the pre-specified sensitivity analysis (Table 2), eight of the nine originally significant regional comparisons remained significant; only tCho/*tCr* attenuated to non-significance (*p* = 0.073). The key findings—the GPC anterior-to-posterior gradient, elevated OC GABA, anterior-dominant mI, and the posterior-dominant tNAA/Asp pattern—were robust to the exclusion of potentially lower-quality spectra, confirming the reliability of the principal regional metabolite profiles.

### 3.6 Normative reference ranges

Table 5 presents the preliminary reference estimates (mean ± 2 SD) for selected key metabolite ratios across the three regions. The coefficient of variation (CV%) indexes inter-individual variability and does not, by itself, measure within-subject reproducibility or measurement precision. The PCC consistently showed the lowest CV% for most metabolites, indicating lower inter-individual variability in that region (for example, NAA/tCr CV was 13.3% in the PCC versus 23.4% in the DACC and 19.5% in the OC); this reflects biological and/or technical homogeneity across individuals rather than measurement reproducibility. Among metabolites, tNAA/tCr and mI/tCr showed the lowest inter-individual variability (CV 10.9–17.6%), whereas Lac/tCr and PCh/tCr showed the highest inter-individual variability (CV 41–72%), consistent with their low signal-to-noise characteristics; of these, Lac also carries high per-metabolite Bayesian quantification uncertainty, whereas PCh’s median Bayesian uncertainty is low (3.3–4.4%; Supplementary Table 6).

**TABLE 5:** Normative reference ranges (mean ± 2 SD) and coefficient of variation for selected key metabolites.

VOI	DACC			PCC			OC		
Met	Mean ± SD	95% RI	CV%	Mean ± SD	95% RI	CV%	Mean ± SD	95% RI	CV%
NAA	1.33 ± 0.31	0.71–1.96	23.4	1.43 ± 0.19	1.05–1.81	13.3	1.64 ± 0.32	1.00–2.28	19.5
tNAA	1.44 ± 0.23	0.97–1.91	16.3	1.54 ± 0.17	1.20–1.87	10.9	1.74 ± 0.28	1.18–2.30	16.0
GABA	0.18 ± 0.07	0.04–0.31	37.7	0.17 ± 0.05	0.08–0.26	27.5	0.25 ± 0.07	0.11–0.40	28.6
mI	1.17 ± 0.20	0.78–1.57	16.9	1.03 ± 0.16	0.72–1.34	15.1	1.01 ± 0.18	0.66–1.37	17.6
GPC	0.21 ± 0.07	0.08–0.34	31.5	0.14 ± 0.04	0.07–0.22	26.9	0.09 ± 0.03	0.02–0.15	37.6
Asp	0.26 ± 0.06	0.15–0.37	21.1	0.21 ± 0.03	0.16–0.27	12.8	0.29 ± 0.07	0.14–0.43	25.9
GSH	0.14 ± 0.07	0.00–0.28	51.3	0.15 ± 0.05	0.05–0.25	34.5	0.11 ± 0.03	0.04–0.17	29.5
Glc	0.36 ± 0.13	0.11–0.61	34.5	0.25 ± 0.08	0.10–0.41	30.8	0.33 ± 0.14	0.04–0.61	43.5

95% RI = 95% reference interval (mean ± 2 SD; lower bound truncated at zero where applicable). CV%, coefficient of variation. Selected key metabolites shown; the full 14-metabolite reference dataset, including observed ranges, is provided in Supplementary Table 4.

## 4 Discussion

This study provided preliminary, cohort-specific multi-regional metabolite reference estimates in cognitively intact older adults. The principal findings were: (1) robust region-specific differences in metabolite distributions—confirmed by the within-subject Friedman test and surviving FDR correction—with a very large effect for GPC (Kendall’s *W* = 0.82) and medium effects for GABA, Asp, and tNAA (*W* = 0.25–0.33); (2) exploratory PCC age associations, most consistently a coordinated aspartate–glutathione decline that survived region-wise FDR correction but not correction across all 42 tests; (3) no significant age-related changes in the occipital cortex; and (4) no significant sex differences. All age-related observations are hypothesis-generating and require confirmation in larger cohorts.

### 4.1 Regional metabolite profiles

The regional metabolite distribution patterns observed in this study are broadly consistent with previous ^1^H-MRS literature. The posterior-dominant gradient of tNAA/tCr, with highest concentrations in OC, aligns with reports by Pouwels and Frahm (1998) and Maudsley et al. (2009), reflecting higher neuronal density in primary sensory cortices. The anterior-dominant distribution of mI/tCr and tCho/tCr is consistent with greater astrocytic content in prefrontal and cingulate regions.

GPC/tCr demonstrated the most striking regional variation (ε^2^ = 0.55), with a clear anterior-to-posterior gradient (DACC > PCC > OC). This pattern, previously reported by Tal et al. (2012), reflects regional differences in membrane phospholipid metabolism and may relate to varying rates of membrane turnover across cortical regions. The elevated GABA concentration in OC relative to DACC and PCC is consistent with the known high density of GABAergic interneurons in visual cortex (Eylers et al., 2016) and has important implications for clinical GABA-MRS studies.

### 4.2 PCC age-related metabolic changes

Although the PCC age associations did not survive FDR correction across all 42 tests—reflecting the statistical-power limitation of a pilot sample (*n* = 20)—the coordinated aspartate and glutathione declines survived region-wise FDR correction (both *q* = 0.044), and the PCC showed the most prominent age associations among the three regions. The convergent decline across biologically related metabolites within a single region provides a coherent, if preliminary, neurobiological narrative. The concurrent nominal decline in PCC glutamate (Glu/*tCr*; ρ = –0.45, *p* = 0.049) is biologically interpretable: glutamate is interconverted with α-ketoglutarate in the tricarboxylic-acid cycle, co-segregating mechanistically with the observed aspartate decline (a malate–aspartate-shuttle substrate).

The strongest age association was observed for Asp/*tCr*. Aspartate participates in mitochondrial energy metabolism through the malate–aspartate shuttle (Moffett et al., 2007), and its decline may reflect age-related mitochondrial dysfunction in the PCC; we note, however, that aspartate is a low-concentration metabolite with relatively high per-measurement uncertainty (Supplementary Table 6), so this association—although it survived region-wise FDR correction—should be regarded as hypothesis-generating. The concurrent decline in GSH/*tCr*, the brain’s primary endogenous antioxidant (quantified with substantially lower uncertainty), is particularly noteworthy, suggesting increased oxidative vulnerability in the aging PCC, consistent with Mandal et al. (2015). The tNAA/*tCr* trend aligns with the widely reported age-related decrease in the neuronal marker NAA (Haga et al., 2009; Cleeland et al., 2019).

The PCC is a core hub of the DMN and one of the most metabolically active brain regions at rest (Buckner et al., 2008). Its high baseline metabolic demand may render it particularly vulnerable to age-related bioenergetic decline. The co-occurring age-related declines in aspartate (mitochondrial metabolism) and glutathione (antioxidant defense)—accompanied by a concordant trend for total NAA (neuronal integrity)—suggest a coherent, if preliminary, pathophysiological pattern that, if confirmed in larger studies, could serve as a candidate multi-metabolite panel for monitoring default-mode-network aging. Taken together, the convergent age-related decline of aspartate (a malate–aspartate-shuttle substrate that indexes mitochondrial bioenergetic capacity) and glutathione (the brain’s principal endogenous antioxidant) within the PCC—both surviving region-wise FDR correction—is consistent with a mitochondria–redox vulnerability in this default-mode-network hub and, although preliminary, warrants confirmation in larger and longitudinal cohorts.

### 4.3 OC as a candidate stable reference region

In contrast to the PCC, the OC showed apparent metabolic stability across the 56–84-year range, with none of the 14 metabolite ratios showing a significant age correlation. This suggestive observation may have clinical relevance: occipital metabolite values could represent a candidate age-independent reference for within-subject comparisons, conceptually analogous to reference regions in PET neuroimaging (Minoshima et al., 1995). Such a within-subject ratio (target region/OC reference) might yield diagnostic metrics that are more robust to inter-individual variation in global metabolite levels. This interpretation remains preliminary and requires validation in larger, independent cohorts; the apparent stability is limited to the 56–84-year range and to cross-sectional data, and whether the OC remains stable at younger ages, longitudinally, or under pathology is unknown. Because total creatine—the denominator of all reported ratios—may itself vary with age and region (see Limitations), an apparently stable ratio does not guarantee a stable underlying concentration.

### 4.4 MMSE and metabolite associations

Age and MMSE scores were not significantly correlated in this cognitively intact cohort (Spearman ρ = –0.28, *p* = 0.23, *n* = 20). Exploratory Spearman analyses (uncorrected) of MMSE–metabolite associations across 42 region × metabolite tests revealed two nominally significant positive correlations in the posterior cingulate cortex (PCC): mI/tCr (ρ = +0.54, *p* = 0.013) and Asp/tCr (ρ = +0.46, *p* = 0.041), with additional trend-level positive associations for Glx/tCr in the PCC (ρ = +0.43, *p* = 0.056) and mI/tCr in the DACC (ρ = +0.38, *p* = 0.099). No correlations were observed in the occipital cortex. None of these associations survived Benjamini–Hochberg correction across the full set of tests (all *q* ≥ 0.56), and the narrow MMSE range (24–30) constrains statistical power. Nonetheless, the consistent positive direction for PCC mI/tCr, Asp/tCr, and Glx/tCr—metabolites linked to glial-osmolyte homeostasis and excitatory–glutamatergic signaling—suggests that even within the cognitively normal range, higher MMSE performance tends to co-vary with greater PCC neurochemical integrity, a hypothesis-generating observation warranting validation in larger samples.

### 4.5 MRS quantification

As an additional check, we directly compared the deep-learning quantification against LCModel (version 6.3) across the entire cohort (all 20 participants, 60 spectra), quantifying the same coil-combined, eddy-current-corrected spectra with the provider’s 3T PRESS TE = 35 ms basis set (Supplementary methods 8, Supplementary Table 7, and Supplementary Figure 3). The two methods showed broad agreement in the rank ordering of metabolites (pooled Pearson *r* = 0.87 across metabolites), with the largest disagreements in metabolites that LCModel itself flagged as unreliable (high CRLB: GABA, aspartate, glucose, lactate, phosphocholine); of these, GABA, aspartate and lactate also carry high deep-learning Bayesian uncertainty (Supplementary Table 6), whereas glucose and phosphocholine are quantified with low deep-learning uncertainty despite LCModel’s high CRLB. Even among reliably-quantified metabolites, several showed systematic scale offsets (tCho, Gln, mI and Glx differed by ≈1.3–2-fold; Supplementary Table 7), so the pooled correlation reflects cross-metabolite ordering rather than per-metabolite interchangeability. Reference values from deep-learning and conventional fitting are therefore not directly interchangeable, and method-specific reference databases are needed.

### 4.6 Limitations

Several limitations warrant consideration. First and most importantly, tissue-segmentation correction of voxel composition (gray-matter/white-matter/CSF partial volume) was not performed; the deep-learning pipeline quantifies metabolite ratios from the acquired spectrum without co-registered structural segmentation. Although voxel volume was held constant (≈8 cm^3^) and placements followed standardized anatomical landmarks, regional and age-related differences in gray/white-matter fractions—which can be substantial across the 56–84-year range owing to selective atrophy—may contribute to apparent regional and age-related metabolite differences (Tal et al., 2012). For the standardized midline placements used here, published data indicate predominantly gray-matter voxels for the DACC and OC and a mixed gray/white composition for the precuneal PCC; these are literature-based expectations, not measured per-voxel fractions, and tissue-corrected quantification is a priority for future work. Second, all values were expressed relative to total creatine (tCr), a convenient but imperfect internal reference that is not necessarily age- or region-invariant, with several studies reporting region-specific age-related changes in creatine (Gruber et al., 2008; Ahlswede et al., 2021; Lind et al., 2020; Mahmoudi et al., 2023). A change in a metabolite/tCr ratio may therefore partly reflect a change in the denominator; water-referenced absolute quantification and institution-specific tCr normative data are needed to disambiguate, and the apparent occipital stability in particular should be re-examined with absolute quantification. Third, regarding comparability to conventional fitting, we quantified the entire cohort’s spectra with LCModel (version 6.3, provider 3T PRESS TE = 35 ms basis; Supplementary methods 8): the deep-learning and LCModel estimates showed broad agreement in metabolite rank ordering (pooled Pearson *r* = 0.87), with the largest disagreements in metabolites that LCModel itself flags as unreliable (high CRLB), although several reliably-quantified metabolites showed systematic ≈1.3–2-fold scale offsets (Supplementary Table 7), so the two methods are not directly interchangeable. For 18 of the 20 participants this used the pipeline’s eddy-current-corrected spectra rather than vendor-native raw data (available for 2 participants, for whom LCModel run directly on the raw DICOM reproduced the result), so a prospective comparison from vendor-native raw acquisitions is a reasonable next step. We agree that the presented spectra have adequate SNR; the deep-learning pipeline was chosen not to rescue poor SNR but for its robustness and native uncertainty quantification. Fourth, the sample of 20 participants, although adequate for large regional effects, provided limited power for age correlations (≈29 needed to detect ρ = 0.50 at 80% power) and was unevenly distributed across decades (56–65, *n* = 7; 66–75, *n* = 4; 76–84, *n* = 9) (Supplementary Figure 2). Because neurometabolite profiles and their age dependence differ across this three-decade span and between typically-aging adults and superagers (Lind et al., 2020; de Godoy et al., 2021), and because brain structural change accelerates in later life (Bethlehem et al., 2022), the single, pooled reference set reported here is preliminary, and age-stratified references await a larger cohort. Fifth, for multiple-comparison control we report both a region-wise FDR correction (three families of 14 tests, reflecting the anatomical independence of the acquisitions) and the more conservative correction across all 42 tests, so age associations can be judged under both schemes; a fully specified linear mixed-effects model with subject-level random intercepts—more efficient than the omnibus tests used here—is a priority for larger cohorts. Finally, this single-center, cross-sectional study used midline voxels only (lateralized differences were not assessed) and scans were not strictly time-of-day matched, so diurnal variation in metabolites such as lactate and glutathione cannot be fully excluded.

### 4.7 Clinical implications and future directions

The preliminary reference estimates established in this study have potential applicability in rehabilitation medicine. Individual patient metabolite values could be compared against the preliminary reference ranges (Table 5) to flag candidate regional metabolic abnormalities in conditions such as stroke, TBI, and mild cognitive impairment. The identification of the OC as a candidate metabolically stable reference region offers a potential within-subject normalization approach that may enhance diagnostic sensitivity, pending validation.

Future studies should: (1) expand and age-stratify the sample for adequate power after FDR correction; (2) incorporate tissue-segmentation correction and water-referenced absolute quantification to complement *tCr*-normalized ratios; (3) adopt longitudinal designs and linear mixed-effects modeling to track within-individual trajectories; and (4) apply the preliminary reference estimates to clinical populations (stroke, TBI, MCI) and extend to multicenter protocols.

## 5 Conclusion

This study established preliminary, deep-learning-based *^1^H*-MRS multi-regional reference estimates for cognitively intact older adults. Robust regional metabolite profiles with large effect sizes were observed—confirmed by within-subject Friedman testing and surviving FDR correction—with GPC showing the strongest regional gradient. The PCC showed suggestive, hypothesis-generating age-related change, most consistently a coordinated aspartate–glutathione decline that survived region-wise FDR correction, requiring confirmation in adequately powered cohorts. The OC emerged as a candidate metabolically stable region whose suitability for age-independent within-subject comparison warrants validation. Given the small pilot sample, the absence of tissue-segmentation correction, and reliance on *tCr* referencing, these estimates are preliminary and provide a quantitative benchmark for future confirmatory work rather than definitive normative values.

## Data Availability

The original contributions presented in this study are included in the article/Supplementary material, further inquiries can be directed to the corresponding author.
