# Supplementary material for: Region-specific brain metabolite reference estimates and exploratory age-related changes in cognitively intact older adults: a preliminary deep-learning 1H-MRS study
Source: Front Aging Neurosci. 2026 Aug 6;18:1896159. doi: 10.3389/fnagi.2026.1896159 (PMC13490163; doi:10.3389/fnagi.2026.1896159)
Supplement: Supplementary file 1 [file Data_Sheet_1.docx]

# Supplementary Material

This supplementary material accompanies the manuscript and provides: (i) FDR-corrected p-values for age–metabolite correlations under both a 42-wide and a region-wise scheme (Table S1); (ii) the secondary Kruskal–Wallis analysis of regional differences (Table S2); (iii) Shapiro–Wilk normality results (Table S3); (iv) complete reference estimates for all metabolite ratios (Table S4); (v) exploratory MMSE–metabolite correlations (Table S5); (vi) per-metabolite Bayesian quantification uncertainties (Table S6); and (vii) a full-cohort comparison of the deep-learning quantification against LCModel (version 6.3) across all 60 spectra (Supplementary Methods S.M.8, Table S7, Supplementary Figure S3). Supplementary Methods and Supplementary Figures S1–S3 are also provided. All values were recomputed on the finalized cohort dataset with verified subject-level demographic linkage; regional comparisons and reference ranges were unchanged, and age-correlation coefficients were refined slightly relative to the original submission.

*All values reported in Tables S1–S5 were computed from the raw cohort data using scipy.stats (v1.12) with a fixed random seed (42) for reproducibility. Friedman statistics in Table S2 are based on all 20 participants, with the three regional voxels paired within each subject. Shapiro–Wilk results in Table S3 are based on the full n = 20 per region.*

## Table S1. Benjamini–Hochberg FDR-corrected p-values for age–metabolite correlations.

Spearman rank correlations (*ρ*) between participant age and metabolite-to-total-creatine (*tCr*) ratios, restricted to associations reaching nominal significance (uncorrected *p* < 0.05). Benjamini–Hochberg FDR-adjusted *q*-values are reported under two schemes: across the full family of 42 tests (14 metabolites × 3 regions) and within each region separately (three families of 14 tests). Under the region-wise scheme, PCC Asp/*tCr* and GSH/*tCr* survived correction (*q* = 0.044).

| **Region / Metabolite** | **ρ** | **p (uncorrected)** | **q (42-wide \| region-wise)** |
| --- | --- | --- | --- |
| PCC Asp/*tCr* | -0.61 | 0.004 | 0.088 \| 0.044 |
| DACC Glc/*tCr* | +0.60 | 0.005 | 0.088 \| 0.072 |
| PCC GSH/*tCr* | -0.59 | 0.006 | 0.088 \| 0.044 |
| DACC Lac/*tCr* | +0.50 | 0.025 | 0.263 \| 0.175 |
| PCC Glu/*tCr* | -0.45 | 0.049 | 0.411 \| 0.228 |

Footnote. *ρ*, Spearman correlation coefficient; *q*-values are reported under two schemes—across all 42 tests and within each region (three families of 14). Under the region-wise scheme, PCC Asp/*tCr* and GSH/*tCr* survived correction (*q* = 0.044); under the 42-wide scheme no association survived (smallest *q* = 0.088). Findings are reported as exploratory and hypothesis-generating, requiring confirmation in larger cohorts. Values rounded to 2-decimal *ρ* and 3-decimal *p* per main-manuscript convention.

## Table S2. Kruskal–Wallis (secondary, concordance) analysis of regional metabolite differences.

Regional differences were analysed primarily with the within-subject Friedman test (main-text Table 2). For concordance, the table below reports the secondary independent-samples Kruskal–Wallis analysis, which does not model within-subject dependence but is widely used; *ε²* is the epsilon-squared effect size and *q* the Benjamini–Hochberg FDR-adjusted *p* across 14 metabolites. The two analyses were concordant for 12 of 14 metabolites; NAA and lactate were significant under Kruskal–Wallis but fell to borderline/non-significant under the more conservative within-subject Friedman test after FDR correction.

| **Metabolite** | **H** | **p** | **q (FDR)** | **ε²** | **KW–Friedman concordance** |
| --- | --- | --- | --- | --- | --- |
| *NAA* | 9.42 | 0.009 | 0.016 | 0.130 | KW-sig only (borderline Friedman) |
| *tNAA* | 13.04 | 0.001 | 0.005 | 0.194 | Concordant |
| *Asp* | 16.84 | <0.001 | 0.002 | 0.260 | Concordant |
| *Gln* | 5.71 | 0.058 | 0.073 | 0.065 | Concordant |
| *Glu* | 0.22 | 0.895 | 0.916 | -0.031 | Concordant |
| *Glx* | 0.17 | 0.916 | 0.916 | -0.032 | Concordant |
| *Glc* | 9.61 | 0.008 | 0.016 | 0.134 | Concordant |
| *mI* | 9.34 | 0.009 | 0.016 | 0.129 | Concordant |
| *tCho* | 7.45 | 0.024 | 0.038 | 0.096 | Concordant |
| *GPC* | 33.56 | <0.001 | <0.001 | 0.554 | Concordant |
| *PCh* | 1.15 | 0.562 | 0.655 | -0.015 | Concordant |
| *GABA* | 15.87 | <0.001 | 0.002 | 0.243 | Concordant |
| *GSH* | 5.89 | 0.053 | 0.073 | 0.068 | Concordant |
| *Lac* | 11.30 | 0.004 | 0.010 | 0.163 | KW-sig only (borderline Friedman) |

Footnote. *H*, Kruskal–Wallis statistic; *p*, uncorrected; *q*, Benjamini–Hochberg FDR-adjusted *p* across 14 metabolites; *ε²*, epsilon-squared effect size. All 20 participants contributed complete measurements across the three regions. The regional ranking pattern is concordant with the primary within-subject Friedman analysis (main-text Table 2) for 12 of the 14 metabolites; NAA and lactate are the two exceptions, each significant under Kruskal–Wallis but borderline/non-significant under the more conservative Friedman test after FDR correction.

## Table S3. Shapiro–Wilk normality test results for metabolite concentrations by brain region.

Shapiro–Wilk tests were performed separately for each metabolite-to-tCr ratio within each brain region (N = 20 per region). Asterisks (*) indicate p < 0.05, suggesting departure from normality; for these ratios, distribution-free 2.5th–97.5th percentile reference intervals are additionally recommended (see Supplementary Methods S.M.5).

| **Metabolite** | **DACC W** | **DACC p** | **PCC W** | **PCC p** | **OC W** | **OC p** |
| --- | --- | --- | --- | --- | --- | --- |
| NAA | 0.98 | 0.964 | 0.97 | 0.728 | 0.96 | 0.573 |
| tNAA | 0.95 | 0.332 | 0.95 | 0.446 | 0.96 | 0.517 |
| Asp | 0.98 | 0.957 | 0.92 | 0.122 | 0.88 | 0.021* |
| Gln | 0.81 | 0.001* | 0.94 | 0.255 | 0.92 | 0.119 |
| Glu | 0.96 | 0.581 | 0.98 | 0.879 | 0.93 | 0.129 |
| Glx | 0.96 | 0.559 | 0.99 | 0.998 | 0.97 | 0.682 |
| Glc | 0.83 | 0.003* | 0.95 | 0.300 | 0.93 | 0.171 |
| mI | 0.98 | 0.875 | 0.85 | 0.006* | 0.96 | 0.463 |
| tCho | 0.97 | 0.768 | 0.93 | 0.124 | 0.94 | 0.268 |
| GPC | 0.97 | 0.693 | 0.89 | 0.024* | 0.93 | 0.167 |
| PCh | 0.96 | 0.450 | 0.95 | 0.360 | 0.95 | 0.330 |
| GABA | 0.94 | 0.197 | 0.95 | 0.440 | 0.97 | 0.659 |
| GSH | 0.95 | 0.339 | 0.97 | 0.655 | 0.97 | 0.852 |
| Lac | 0.90 | 0.049* | 0.93 | 0.153 | 0.92 | 0.087 |

*Footnote. W, Shapiro–Wilk test statistic; p, two-sided p-value for the null hypothesis of normality. Computed using scipy.stats.shapiro (v1.12). Asterisks (*p < 0.05) indicate ratios for which the mean ± 2 SD reference interval should be supplemented with distribution-free percentile-based reference intervals.*

## Table S4. Region-specific reference estimates for all 14 quantified metabolite ratios.

For each metabolite-to-total-creatine (tCr) ratio, region-specific mean ± SD, observed range (minimum–maximum), 95% reference interval (mean ± 2 SD, lower bound truncated at zero), and coefficient of variation (CV%) are reported across DACC, PCC, and OC (n = 20 per region). Reference intervals are intended as preliminary cohort-specific estimates for evaluating individual metabolite values in clinical and research populations; intervals for non-normally distributed ratios (see Table S3) should be interpreted with caution, and distribution-free percentile-based intervals may be preferable in those cases.

| **Metabolite** | **Region** | **Mean ± SD** | **Range** | **95% RI (mean ± 2 SD)** | **CV (%)** |
| --- | --- | --- | --- | --- | --- |
| NAA | DACC | 1.33 ± 0.31 | 0.77–1.98 | 0.71–1.96 | 23.4 |
|  | PCC | 1.43 ± 0.19 | 1.00–1.81 | 1.05–1.81 | 13.3 |
|  | OC | 1.64 ± 0.32 | 1.14–2.43 | 1.00–2.28 | 19.5 |
| tNAA | DACC | 1.44 ± 0.23 | 1.08–1.99 | 0.97–1.91 | 16.3 |
|  | PCC | 1.54 ± 0.17 | 1.18–1.88 | 1.20–1.87 | 10.9 |
|  | OC | 1.74 ± 0.28 | 1.34–2.44 | 1.18–2.30 | 16.0 |
| Asp | DACC | 0.26 ± 0.06 | 0.16–0.37 | 0.15–0.37 | 21.1 |
|  | PCC | 0.21 ± 0.03 | 0.17–0.29 | 0.16–0.27 | 12.8 |
|  | OC | 0.29 ± 0.07 | 0.20–0.46 | 0.14–0.43 | 25.9 |
| Gln | DACC | 0.35 ± 0.15 | 0.17–0.87 | 0.04–0.66 | 43.6 |
|  | PCC | 0.29 ± 0.06 | 0.18–0.39 | 0.17–0.42 | 21.3 |
|  | OC | 0.35 ± 0.07 | 0.23–0.44 | 0.21–0.49 | 20.1 |
| Glu | DACC | 1.17 ± 0.25 | 0.68–1.58 | 0.66–1.68 | 21.7 |
|  | PCC | 1.19 ± 0.15 | 0.92–1.52 | 0.90–1.49 | 12.5 |
|  | OC | 1.18 ± 0.19 | 0.92–1.63 | 0.79–1.57 | 16.5 |
| Glx | DACC | 1.52 ± 0.31 | 0.99–2.33 | 0.90–2.15 | 20.5 |
|  | PCC | 1.49 ± 0.18 | 1.14–1.85 | 1.13–1.84 | 11.9 |
|  | OC | 1.53 ± 0.24 | 1.15–2.07 | 1.05–2.01 | 15.7 |
| Glc | DACC | 0.36 ± 0.13 | 0.19–0.77 | 0.11–0.61 | 34.5 |
|  | PCC | 0.25 ± 0.08 | 0.11–0.37 | 0.10–0.41 | 30.8 |
|  | OC | 0.33 ± 0.14 | 0.04–0.67 | 0.04–0.61 | 43.5 |
| mI | DACC | 1.17 ± 0.20 | 0.79–1.64 | 0.78–1.57 | 16.9 |
|  | PCC | 1.03 ± 0.16 | 0.54–1.34 | 0.72–1.34 | 15.1 |
|  | OC | 1.01 ± 0.18 | 0.64–1.34 | 0.66–1.37 | 17.6 |
| tCho | DACC | 0.52 ± 0.25 | 0.12–1.03 | 0.02–1.03 | 48.0 |
|  | PCC | 0.39 ± 0.12 | 0.21–0.66 | 0.15–0.64 | 31.5 |
|  | OC | 0.34 ± 0.18 | 0.08–0.80 | 0.00–0.70 | 54.5 |
| GPC | DACC | 0.21 ± 0.07 | 0.11–0.34 | 0.08–0.34 | 31.5 |
|  | PCC | 0.14 ± 0.04 | 0.09–0.24 | 0.07–0.22 | 26.9 |
|  | OC | 0.09 ± 0.03 | 0.02–0.15 | 0.02–0.15 | 37.6 |
| PCh | DACC | 0.31 ± 0.20 | 0.01–0.71 | 0.00–0.71 | 63.0 |
|  | PCC | 0.25 ± 0.10 | 0.08–0.44 | 0.04–0.46 | 41.6 |
|  | OC | 0.25 ± 0.16 | 0.02–0.65 | 0.00–0.57 | 63.7 |
| GABA | DACC | 0.18 ± 0.07 | 0.09–0.36 | 0.04–0.31 | 37.7 |
|  | PCC | 0.17 ± 0.05 | 0.10–0.26 | 0.08–0.26 | 27.5 |
|  | OC | 0.25 ± 0.07 | 0.12–0.40 | 0.11–0.40 | 28.6 |
| GSH | DACC | 0.14 ± 0.07 | 0.04–0.32 | 0.00–0.28 | 51.3 |
|  | PCC | 0.15 ± 0.05 | 0.07–0.27 | 0.05–0.25 | 34.5 |
|  | OC | 0.11 ± 0.03 | 0.05–0.17 | 0.04–0.17 | 29.5 |
| Lac | DACC | 0.27 ± 0.19 | 0.04–0.77 | 0.00–0.66 | 71.4 |
|  | PCC | 0.11 ± 0.06 | 0.02–0.25 | 0.00–0.23 | 60.3 |
|  | OC | 0.14 ± 0.09 | 0.02–0.29 | 0.00–0.31 | 62.1 |

Footnote. Mean ± SD computed across all n = 20 spectra per region. Range = observed minimum–maximum. 95% RI = mean − 2 SD to mean + 2 SD (lower bound truncated to zero where mean − 2 SD < 0, because metabolite concentration ratios cannot be negative); for ratios flagged as non-normal in Table S3 (Shapiro–Wilk p < 0.05), distribution-free percentile reference intervals are recommended in future work. CV, coefficient of variation = SD/mean × 100. DACC, dorsal anterior cingulate cortex; PCC, posterior cingulate cortex; OC, occipital cortex.

## Table S5. Exploratory MMSE–metabolite Spearman correlations.

Spearman rank correlations between Mini-Mental State Examination (MMSE) scores and metabolite-to-total-creatine (tCr) ratios were computed for all 14 metabolites in each of the three regions (42 tests; n = 20). Only associations reaching nominal significance (uncorrected p < 0.05) or trend level (0.05 ≤ p ≤ 0.10) are listed; no association survived Benjamini–Hochberg FDR correction.

| Region | Metabolite | ρ | p (uncorrected) | pFDR |
| --- | --- | --- | --- | --- |
| PCC | mI/tCr | +0.54 | 0.013 | 0.56 |
| PCC | Asp/tCr | +0.46 | 0.041 | 0.73 |
| PCC | Glx/tCr | +0.43 | 0.056 | 0.73 |
| DACC | mI/tCr | +0.38 | 0.099 | 0.73 |

Footnote. ρ, Spearman correlation coefficient; pFDR, Benjamini–Hochberg adjusted p-value across all 42 MMSE–metabolite tests. Positive ρ indicates higher metabolite ratio with higher MMSE. No association survived FDR correction (all pFDR ≥ 0.56); these findings are exploratory and hypothesis-generating given the narrow MMSE range (24–30).

## Supplementary Methods

### S.M.1 CNN architecture and quantification pipeline

Metabolite quantification was performed using the convolutional neural network (CNN) framework developed and validated by Lee and Kim (2019, 2020, 2022). Briefly, the network consists of a 1-D encoder–decoder fully-convolutional architecture with seven convolutional blocks (kernel sizes 3–7, feature maps 32–256), batch normalization, and exponential linear unit activations, followed by a fully-connected regression head outputting relative concentrations for the target metabolites. The network was trained on approximately 10 million in silico brain spectra generated with a density-matrix formalism, incorporating realistic noise, line broadening, frequency/phase jitter, and baseline distortions across a wide range of SNR (3–120) and linewidth (3–30 Hz) conditions, to match in vivo 3T single-voxel PRESS acquisition (TR/TE = 2000/35 ms).

### S.M.2 Quality control thresholds

The deep-learning pipeline produced two quality metrics: a spectrum-level reconstruction goodness-of-fit (R²) between the input and the network-reconstructed model spectrum, and a per-metabolite Bayesian relative uncertainty from Monte-Carlo-dropout sampling (expressed as a percentage of the estimated amplitude; Table S6). Spectra with R² < 0.70 were flagged for review. All 60 spectra were included in the primary analyses to preserve the within-subject design; the three flagged DACC spectra (5.0%, all R² < 0.70) were removed only in a pre-specified sensitivity analysis (main-text Table 4), which reproduced the principal regional findings (all except tCho, which attenuated to non-significance, p = 0.073). No separate LCModel-derived CRLB was computed because quantification did not use linear-combination fitting; the Bayesian relative uncertainty (Table S6) is the pipeline-native analogue of the CRLB. The R² ≥ 0.70 flag follows conventions in the deep-learning MRS literature (Lee and Kim, 2019).

### S.M.3 Friedman repeated-measures procedure

To model the repeated-measures structure of the design (three brain regions per participant), the Friedman test was computed as a non-parametric alternative to one-way repeated-measures ANOVA for each metabolite ratio across the three regions (DACC, PCC, OC). The test statistic was computed as χ² = [12 / (N × k × (k + 1))] × ΣRj² − 3 × N × (k + 1), with N = 20 participants and k = 3 regions. Kendall's W = χ² / [N × (k − 1)] was reported as the standardized effect size. Post-hoc pairwise comparisons used Dunn's test with Benjamini–Hochberg correction.

### S.M.4 False discovery rate correction

Spearman correlations between age and each metabolite-to-*tCr* ratio were computed for all 14 metabolites in each of the 3 regions, yielding 42 hypothesis tests. The Benjamini–Hochberg (1995) linear step-up procedure was applied both across the full family of 42 tests and, separately, within each region (three families of 14 tests). The region-wise scheme reflects the anatomically distinct nature of the three acquisitions and was applied at the reviewers' suggestion; both schemes are reported in Table S1. Implemented via statsmodels.stats.multitest.multipletests (method='fdr_bh').

### S.M.5 Normality testing and reference-interval construction

Shapiro–Wilk tests (scipy.stats.shapiro) were computed for each metabolite-to-tCr ratio within each brain region. Reference intervals were constructed primarily as mean ± 2 SD (approximating 95% coverage under normality) where the null hypothesis of normality was not rejected (p ≥ 0.05). For ratios rejecting normality, empirical 2.5th–97.5th percentile intervals were used as distribution-free reference intervals in addition to the parametric mean ± 2 SD interval.

### S.M.6 Software environment

All statistical analyses were performed in Python 3.12 using NumPy 1.26, pandas 2.2, SciPy 1.12 (Kruskal–Wallis, Friedman, Shapiro–Wilk, Wilcoxon, Spearman, bootstrap), scikit-posthocs 0.9 (Dunn's test with Benjamini–Hochberg correction), and statsmodels 0.14 (Benjamini–Hochberg FDR). Plots were generated with Matplotlib 3.8 and seaborn 0.13. Random-number seeds were fixed at 42 for all bootstrap and permutation procedures to ensure reproducibility.

## Sensitivity Analysis Outputs

Two sensitivity analyses evaluated the robustness of the primary findings. First, excluding the three DACC spectra with R² < 0.70 (n = 17 for the DACC) and re-running the regional comparison reproduced the principal regional findings, with only tCho attenuating to non-significance (p = 0.073; main-text Table 4). Second, the primary within-subject Friedman test (main-text Table 2) and the secondary Kruskal–Wallis test (Table S2) were concordant for 12 of the 14 metabolites; NAA and lactate reached significance under Kruskal–Wallis but not under the more conservative within-subject Friedman analysis after FDR correction, confirming that the within-subject dependence structure did not alter the qualitative inferences regarding regional metabolic heterogeneity. Together, these analyses support the robustness of the reported preliminary reference estimates and exploratory age-related trends.

## Table S6. Per-metabolite Bayesian quantification uncertainty by brain region.

Median per-metabolite Bayesian relative uncertainty (Monte-Carlo-dropout standard deviation as a percentage of the estimated amplitude) across n = 20 spectra per region. This pipeline-native metric is analogous in role to the Cramér–Rao lower bound of conventional linear-combination fitting. Well-resolved metabolites (uncertainty ≈5% or less) are quantified with high confidence; low-concentration metabolites (GABA, Asp, GSH, Lac) show higher uncertainty and are interpreted with corresponding caution.

| **Metabolite** | **DACC (%)** | **PCC (%)** | **OC (%)** |
| --- | --- | --- | --- |
| *NAA* | 0.4 | 0.5 | 0.4 |
| *tNAA* | 0.3 | 0.3 | 0.2 |
| *Asp* | 14.1 | 38.7 | 11.5 |
| *Gln* | 9.6 | 13.7 | 6.0 |
| *Glu* | 1.5 | 1.8 | 1.6 |
| *Glx* | 0.8 | 0.9 | 0.7 |
| *Glc* | 2.2 | 3.5 | 1.9 |
| *mI* | 1.2 | 1.7 | 1.4 |
| *tCho* | 0.9 | 1.5 | 1.9 |
| *GPC* | 3.8 | 8.0 | 16.5 |
| *PCh* | 3.3 | 4.4 | 3.9 |
| *GABA* | 18.7 | 40.2 | 6.2 |
| *GSH* | 7.1 | 8.6 | 6.3 |
| *Lac* | 50.9 | 137.1 | 105.2 |

*Footnote. Values are medians across 20 spectra per region. tNAA, Glx, and tCho are composite estimates. DACC, dorsal anterior cingulate cortex; PCC, posterior cingulate cortex; OC, occipital cortex.*

## S.M.7 Published cross-method validation against LCModel

A prospective, same-cohort comparison against LCModel was outside the scope of this application study (see main-text Limitations). We therefore summarize the published validation of the exact deep-learning pipeline used here. Lee and Kim (2019) compared the CNN against LCModel on *in vivo* human 3T brain spectra (*n* = 40 spectra from five healthy volunteers acquired at substantially different SNR): metabolite concentrations agreed with LCModel for major metabolites, and the CNN estimates were less variable across SNR (≈10% or less) than the LCModel estimates, indicating greater robustness to acquisition quality. On simulated test data the pipeline achieved a mean absolute percentage error of 12.5% ± 4.4% across aspartate, creatine, GABA, glucose, glutamine, glutamate, glutathione, *myo*-inositol, NAA, phosphocreatine, phosphorylethanolamine, and taurine. Lee and Kim (2022) added Bayesian uncertainty estimation via Monte-Carlo dropout (50 samples): predicted uncertainties correlated with ground-truth error (*r* = 0.72–0.94; mean 0.83 ± 0.06, *p* < 0.001) and, on degraded *in vivo* spectra, tracked the true variation in metabolite content at least as closely as the Cramér–Rao lower bounds from LCModel (0.938 ± 0.019 vs. 0.881 ± 0.057; the difference was not statistically significant, *p* = 0.115). These results support the reliability of the pipeline for the well-resolved metabolites emphasized in the present study while motivating a future same-cohort head-to-head comparison. An additional validation in rat brain at 9.4 T (Lee and Kim, 2020) is methodologically related but was performed on animal data and is not used here as human-brain evidence.

# Supplementary Figures

Supplementary Figure S1. Per-metabolite Bayesian quantification uncertainty (Monte-Carlo-dropout) by region, with a 20% reference line marking the conventional CRLB caution threshold. Well-resolved metabolites (NAA, tNAA, Glu, Glx, mI, tCho, Glc) fall well below 20%, whereas low-concentration metabolites (Asp, GABA, Lac) exceed it in some regions.

Supplementary Figure S2. (A) Age distribution of the cohort by decade band (56–65 years, *n* = 7; 66–75 years, *n* = 4; 76–84 years, *n* = 9). (B) PCC aspartate/*tCr* across decade bands, illustrating the age-related decline underlying the correlation analysis.

## S.M.8 Direct comparison against LCModel

To address the reviewers' request, we directly compared the deep-learning quantification against LCModel (Provencher; version 6.3-1R), the field-standard linear-combination-fitting package, across the entire cohort. For all 20 participants (60 spectra spanning the DACC, PCC and OC), the coil-combined, eddy-current-corrected spectra—the conventional pre-quantification data underlying the deep-learning analysis—were exported and independently quantified with LCModel using the provider's own 3T PRESS TE = 35 ms basis set (press_te35_3t_v3), which includes a full macromolecule/lipid model, with eddy-current correction and water scaling from the water reference. This gives a like-for-like comparison in which the deep-learning pipeline and LCModel quantify the same input spectrum. For the two participants with archived vendor DICOM, running LCModel directly on the DICOM reproduced the exported-spectrum result (e.g., DACC NAA/tCr 1.33 vs 1.33), confirming the export. Metabolite/total-creatine ratios and LCModel Cramér–Rao lower bounds (CRLB, %SD) were extracted and compared with the deep-learning values.

For the metabolites that LCModel quantified reliably (median CRLB ≤ 20%: NAA, tNAA, tCho, GPC, Glu, Glx, mI, Gln, GSH), the deep-learning and LCModel metabolite/tCr estimates showed broad agreement in rank ordering across the cohort: pooled Pearson r = 0.87, Lin's concordance correlation coefficient = 0.87, bias = +0.03, mean absolute error = 0.24 metabolite/tCr units (n = 540 metabolite × voxel comparisons; Table S7, Figure S3). Across all 14 metabolites the correlation was r = 0.90 (n = 840). The largest disagreements were in metabolites that LCModel itself flagged as unreliable (median CRLB ≥ 60%; Figure S3B): GABA, lactate and aspartate, which also carry high deep-learning Bayesian uncertainty (Table S6), and glucose and phosphocholine, which have low deep-learning uncertainty despite LCModel's high CRLB. Even among reliably-quantified metabolites, several showed systematic scale offsets (tCho, Gln, mI and Glx differed by ≈1.3–2-fold; Table S7), and per-metabolite correlations were modest, so the pooled correlation indexes cross-metabolite rank ordering rather than per-metabolite interchangeability. This comparison supports broad comparability of the deep-learning quantification to LCModel while confirming that the two methods are not directly interchangeable and that low-CRLB reliability does not guarantee identical values. An independent open-source linear-combination fit (FSL-MRS, with a simulated ideal-PRESS basis) gave concordant results. A remaining limitation is that, for 18 of the 20 participants, LCModel was applied to the pipeline's eddy-current-corrected spectra rather than to vendor-native raw acquisitions (available for 2 participants, for whom the raw-DICOM fit reproduced the result); a prospective comparison from vendor-native raw data in a new cohort is a reasonable next step.

## Table S7. Deep-learning (CNN) vs. LCModel 6.3 metabolite/tCr estimates across the full cohort (n = 20; 60 spectra), with LCModel CRLB.

| **Metabolite** | **CNN–DL (mean ± SD)** | **LCModel 6.3 (mean ± SD)** | **LCModel CRLB (median %SD)** |
| --- | --- | --- | --- |
| NAA | 1.47 ± 0.30 | 1.29 ± 0.17 | 3% |
| tNAA | 1.57 ± 0.26 | 1.43 ± 0.16 | 3% |
| tCho | 0.42 ± 0.21 | 0.22 ± 0.06 | 4% |
| GPC | 0.15 ± 0.07 | 0.22 ± 0.06 | 4% |
| Glu | 1.18 ± 0.20 | 1.30 ± 0.15 | 6% |
| Glx | 1.51 ± 0.25 | 1.98 ± 0.21 | 6% |
| mI | 1.07 ± 0.19 | 0.76 ± 0.11 | 5% |
| Gln | 0.33 ± 0.11 | 0.68 ± 0.15 | 16% |
| GSH | 0.13 ± 0.06 | 0.19 ± 0.04 | 20% |
| GABA | 0.20 ± 0.07 | 0.11 ± 0.05 | 63% |
| Lac | 0.17 ± 0.14 | 0.10 ± 0.08 | 67% |
| Asp | 0.25 ± 0.06 | 0.11 ± 0.10 | 85% |
| Glc | 0.32 ± 0.13 | 0.08 ± 0.07 | 88% |
| PCh | 0.27 ± 0.16 | 0.01 ± 0.03 | 999% |

Footnote. Values are mean ± SD of metabolite/tCr across all 60 spectra (20 participants × DACC/PCC/OC). LCModel version 6.3-1R with the provider's 3T PRESS TE = 35 ms basis set, applied to the coil-combined, eddy-current-corrected spectra underlying the deep-learning analysis. CRLB, Cramér–Rao lower bound (median %SD); metabolites with CRLB ≤ 20% are reliably quantified (GSH is borderline at 20%). Composite ratios: tNAA = NAA+NAAG, tCho = GPC+PCh, Glx = Glu+Gln, tCr = Cr+PCr. Of the high-CRLB metabolites (GABA, Lac, Asp, Glc, PCh), GABA, Lac and Asp also carry high deep-learning Bayesian uncertainty (Table S6), whereas Glc and PCh have low deep-learning uncertainty.

Supplementary Figure S3. Full-cohort comparison of deep-learning quantification against LCModel 6.3 (n = 20; 60 spectra; Provencher 3T PRESS TE = 35 ms basis). (A) CNN–DL vs. LCModel metabolite/tCr: metabolites LCModel quantifies reliably (median CRLB ≤ 20%; circles) span the identity line (pooled Pearson r = 0.87), whereas high-CRLB metabolites (triangles) diverge. (B) LCModel CRLB by metabolite; of the metabolites LCModel flags as unreliable (≥ 20%), GABA, aspartate and lactate also have the highest deep-learning Bayesian uncertainty (Table S6), whereas glucose and phosphocholine have high LCModel CRLB but low deep-learning uncertainty.

## Supplementary References

All references cited in this Supplementary Material are listed in the main manuscript Reference list
